# The Herbal Drug *Melampyrum pratense* L. (Koch): Isolation and Identification of Its Bioactive Compounds Targeting Mediators of Inflammation

**DOI:** 10.1155/2013/395316

**Published:** 2013-02-26

**Authors:** S. Vogl, A. G. Atanasov, M. Binder, M. Bulusu, M. Zehl, N. Fakhrudin, E. H. Heiss, P. Picker, C. Wawrosch, J. Saukel, G. Reznicek, E. Urban, V. Bochkov, V. M. Dirsch, B. Kopp

**Affiliations:** ^1^Department of Pharmacognosy, University of Vienna, 14 Althanstrasse, 1090 Vienna, Austria; ^2^Department of Vascular Biology and Thrombosis Research, Medical University of Vienna, 17 Schwarzspanierstrasse, 1090 Vienna, Austria; ^3^Department of Pharmaceutical Biology, Faculty of Pharmacy, Universitas Gadjah Mada, Sekip Utara, Yogyakarta 55281, Indonesia; ^4^Department of Medicinal Chemistry, University of Vienna, 14 Althanstrasse, 1090 Vienna, Austria

## Abstract

*Melampyrum pratense* L. (Koch) is used in traditional Austrian medicine for the treatment of different inflammation-related conditions. In this work, we show that the extracts of *M. pratense* stimulated peroxisome proliferator-activated receptors- (PPARs-)**α** and -**γ** that are well recognized for their anti-inflammatory activities. Furthermore, the extract inhibited the activation of the proinflammatory transcription factor NF-**κ**B and induction of its target genes interleukin-8 (IL-8) and E-selectin *in vitro*. Bioassay-guided fractionation identified several active flavonoids and iridoids including melampyroside and mussaenoside and the phenolic compound lunularin that were identified in this species for the first time. The flavonoids apigenin and luteolin were distinguished as the main components accountable for the anti-inflammatory properties. Apigenin and luteolin effectively inhibited tumor necrosis factor **α** (TNF-**α**)-induced NF-**κ**B-mediated transactivation of a luciferase reporter gene. Furthermore, the two compounds dose-dependently reduced IL-8 and E-selectin protein expression after stimulation with lipopolysaccharide (LPS) or TNF-**α** in endothelial cells (ECs). The iridoids melampyroside and mussaenoside prevented the elevation of E-selectin in LPS-stimulated ECs. Lunularin was found to reduce the protein levels of the proinflammatory mediators E-selectin and IL-8 in ECs in response to LPS. These data validate the ethnomedical use of *M. pratense* for the treatment of inflammatory conditions and point to the constituents accountable for its anti-inflammatory activity.

## 1. Introduction

*Melampyrum pratense* L. (Koch) or common cow-wheat belongs to the family of Orobanchaceae and is a herbaceous flowering plant. In Austrian ethnomedicine, the dried herbal drug has been used externally in a pillow or as tea to treat gout and rheumatism [1]. In Romanian traditional medicine, *Melampyrum* species are used to treat rheumatic disorders and skin infections [2]. In England, the plant was recommended as decoction for kidney ailments [3]. Altogether, the applications of this plant in traditional medicine strongly suggest anti-inflammatory activity. 

This work, therefore, aims at validating the anti-inflammatory activity of *M. pratense* and identifying the compounds responsible for this effect by means of bioassay-guided fractionation. To this end, we applied a panel of functional and target-oriented cell models in order to identify constituents of *M. pratense* that abolish TNF-*α* or LPS-induced expression of proinflammatory adhesion molecules (E-selectin) or chemokines (IL-8), inhibit the nuclear factor *κ*-light-chain enhancer of activated B cells (NF-*κ*B) pathway, or activate peroxisome-proliferator-activated receptors (PPARs), thus interfering with different steps of the complex inflammatory process.

Although there are various molecular mediators involved in the development of the inflammatory response, many proinflammatory pathways converge at the point of the activation of NF-*κ*B [4–6]. It regulates the expression of an array of inflammatory response genes such as cytokines (e.g., TNF-*α*, IL-1*β*, IL-6/10/12, and IFN-*γ*), chemokines (e.g., monocyte chemotactic protein-1 (MCP-1), IL-8), enzymes producing inflammatory mediators (e.g., COX-2, 5-lipoxygenase, and inducible nitric oxide synthase), adhesion molecules (e.g., E-selectin, ICAM-1), and enzymes degrading extracellular matrix (e.g., matrix metallopeptidase 9). Hence, targeting different steps of the NF-*κ*B signaling cascade is a promising approach to combat different inflammatory conditions [7]. E-selectin belongs to the endothelial adhesion molecules and plays an important role in recruiting leukocytes to the site of inflammation as it recognizes and binds to sialylated carbohydrates present on their surface proteins. Interleukin-8 (IL-8), a prototype of CXC chemokines, mainly acts as a neutrophil chemoattractant; its production is induced by TNF-*α*, LPS, and IL-1 [8, 9]. Another ubiquitously expressed class of transcription factors that are relevant regulators of the inflammatory response is the one of the nuclear receptors [10]. Recently, much attention has been drawn to the PPARs, which control the expression of inflammatory and metabolic genes in macrophages and lymphocytes [11–13]. PPARs are ligand-activated transcription factors exerting important functions in lipid and glucose metabolism but also regulating inflammation. PPAR*α*, which is highly expressed in the vascular wall, skeletal muscle, heart, liver, and kidney, is mainly involved in the regulation of lipid catabolism [14]. Its activation was also shown to inhibit proliferation [15, 16] and to modulate the inflammatory response of vascular smooth muscle cells (VSMCs) [17]. The activation of the other PPAR subtype studied in this work, PPAR*γ*, increases insulin sensitivity and contributes to the regulation of genes involved in inflammation, hypertension, and atherosclerosis [10, 18–20]. 

The chemical composition of *M. pratense* is very scarcely described in the literature. The aucubin content was determined as 5.11% [21]. 23 phenolic compounds, including 17 flavonoids and 6 phenol carboxylic acids, have been identified by two-dimensional paper chromatography [22, 23]. Suomi et al. (2001) quantified iridoid glycosides in *M. pratense* using optimal on-line combination of partial filling micellar electrokinetic chromatography and electrospray ionization mass spectrometry (ESI-MS) and found aucubin at a concentration of ca. 100 mg/L and traces of catalpol [24]. 

In this work, we performed detailed chemical analysis of *M. pratense* and tested a number of plant constituents in cell-based *in vitro* assays for PPAR*α* and PPAR*γ* activation, NF-*κ*B inhibition, and downregulation of TNF-*α*- or LPS-stimulated expression of E-selectin and IL-8. Our data clearly demonstrate *in vitro* anti-inflammatory activity of *M. pratense *extracts and their individual components.

## 2. Materials and Methods

### 2.1. Materials

Methanol (MeOH) and acetonitrile (MeCN) were of p.a. grade for extraction and of HPLC grade for HPLC analyses (VWR, Vienna, Austria). Ethyl acetate (EtOAc) p.a., chloroform GPR Rectapur (p.a.), dichloromethane (DCM) AnalR Normapur (p.a.), *n*-hexane p.a., and toluol p.a. were obtained from VWR (Vienna, Austria). Formic acid > 98% p.a. was purchased from Carl Roth (Karlsruhe, Germany). Reference compounds apigenin (batch number 050818082968, >99% purity) and luteolin (batch number 020717205864, >99% purity) were from Extrasynthese (Lyon, France); chrysoeriol (batch number 47792261, >98% purity) and diosmetin (batch number 30680794, >98% purity) were from Carl Roth GmbH und CoKG (Karlsruhe, Germany); aucubin (batch number 3495, >99% purity), catalpol (batch number 1344, >99% purity), loganin (batch number 5970, >99% purity), and loganic acid (batch number 3518, >99% purity) were from Phytolab (Vestenbergsgreuth, Germany), and mussaenosidic acid (batch number AD111810-01, 81% purity) was from AnalytiCon Discovery GmbH (Potsdam, Germany).

For bioactivity evaluation, all indicated compounds or dried extracts were reconstituted in dimethyl sulfoxide (DMSO), aliquoted, and stored at −20°C until use.

### 2.2. Plant Material

The aerial parts of *Melampyrum pratense* were field collected at Neustift am Walde, Vienna, Austria. A voucher specimen (MP16072008) is deposited at the Department of Pharmacognosy, University of Vienna, Austria. 

### 2.3. Extraction and Fractionation

183 g of dried and powdered plant material was first processed with the nonpolar solvent DCM yielding 3.7 g (2.0% yield) extract and subsequently with the polar solvent MeOH yielding 28.4 g (15.5% yield) using an accelerated solvent extractor ASE200 (Thermo Scientific Austria GmbH, Vienna, Austria). The instrument was equipped with 22 mL stainless steel extraction cells and 60 mL glass collection bottles. The extraction was performed at 40°C and 150 bar using 3 extraction cycles, 5 min heat-up time, 2 min static time, 10% flush volume, and 60 sec nitrogen purge. 

Subsequently, tannins and chlorophylls were excluded from polar and nonpolar extracts, respectively, in order to avoid possible interferences with the assays and to increase the relative amount of the active compounds. The tannins were removed using liquid-liquid partitions between CHCl_3_ and mixtures of MeOH/H_2_O [25], leading to a loss of 95% of the original MeOH extract yielding 1.4 g (4.9% of the MeOH extract, 0.7% of the crude drug). Chlorophyll was removed from the DCM extract by liquid-liquid-partition between DCM and MeOH : H_2_O (1 : 1) yielding 647.5 mg (17.5% of the DCM extract, 0.3% of the crude drug). In detail, 1 g extract was dissolved in 150 mL DCM, 150 mL MeOH : H_2_O (1 : 1) was added, and DCM was evaporated under reduced pressure (800–600 mbar) at 40°C. Consequently, insoluble chlorophyll precipitated in the methanol-water phase and could be filtered.

Solid phase extraction (SPE) on 60cc Bond Elut C_18_ cartridges (Varian, Harbor City, CA, USA) was used for further fractionation of the purified extracts. The cartridges were prewashed with H_2_O and MeOH and conditioned with 30% MeOH/H_2_O. After the application of the extract, successive elution with different mixtures of MeOH/H_2_O was performed under reduced pressure (flow rate: *≈*2 drops/sec), to obtain three fractions of decreasing polarity (30%, 70%, and 100% methanolic SPE subfractions). 

178 mg of the 30% MeOH/H_2_O SPE fraction of chlorophyll-depleted DCM extract was subjected to column chromatography (CC) on Silica Gel 60 (particle size of 0.063–0.200 mm, Merck, Darmstadt, Germany). The column was first eluted with EtOAc : MeOH : H_2_O 17 : 3 : 1, and 265 fractions were collected. Afterwards the column was eluted with EtOAc : MeOH : H_2_O 17 : 4 : 1. As result of the two elution steps, overall 6 main combined fractions were obtained (30/1–30/6). Fraction 30/1 (12.7 mg) mainly contained aucubin. From subfractions 30/3 (19.1 mg, 10.7% of original SPE fraction), melampyroside was isolated by SPE on C_18_ material upon elution with H_2_O and then MeOH : H_2_O 1 : 9 and 2 : 8. From fraction 30/5 (49.1 mg, 27.5% of original SPE fraction), mussaenoside was purified using SPE on C_18_ material upon elution with H_2_O and then MeOH : H_2_O 1 : 9 and 2 : 8.

239 mg of the 70% MeOH/H_2_O SPE fraction of the chlorophyll-depleted DCM extract was subjected to CC on Silica Gel 60 (particle size of 0.063–0.200 mm, Merck, Darmstadt, Germany) using a mobile phase of CHCl_3_ : MeOH : H_2_O 90 : 3.5 : 0.2 and 85 : 8 : 0.5 from fraction 675 on. Fractions were combined to 13 main fractions (70/1–70/13). Fractions 70/4 (2.5 mg, 1.1% of original SPE fraction) and 70/6 (9.5 mg, 4.0% of original SPE fraction) mainly consisted of lunularin. Fraction 70/5 (23.4 mg, 9.8% of original SPE fraction) contained pure lunularin (structure clarified via LC-MS and NMR). 

### 2.4. HPLC Analysis

An HPLC instrument from Shimadzu (Kyoto, Japan) equipped with a CBM-20A system controller, a DGU-20A5 membrane degasser, an LC-20AD solvent delivery unit, an SIL-20AC HT autosampler, a CTO-20AC column oven, an SPD-M20A photodiode array detector, and a low-temperature evaporative light scattering detector (ELSD-LT) was used for HPLC-DAD/ELSD experiments. Data analysis was conducted with the chromatography software LCsolution Ver.1.2 (Shimadzu). Chromatographic separation was performed on an Acclaim 120 C_18_ reversed-phase column (150 mm × 2.1 mm i.d., 3 *μ*m) equipped with an Acclaim 120 C_18_ guard column (10 mm × 2.1 mm i.d., 5 *μ*m) from Dionex (Germering, Germany), at 35°C and a flow rate of 0.4 mL/min. Water modified with formic acid (pH = 2.6) and MeCN were used as mobile phase A and B, respectively. For the methanolic extract, gradient elution was performed as follows: start isocratic at 2% of B for 5 min, followed by 2%–10% of B in 11 min, 10%–20% of B in 24 min, 20%–50% of B in 20 min, and 50%–95% of B in 5 min. For all other extracts, the following gradient was used: start isocratic at 2% of B for 5 min, followed by 2%–15% of B in 5 min, 15%–21% of B in 3 min, 21%–35% of B in 27 min, and 35%–95% of B in 10 min. The injection volume was 5 *μ*L for all samples. The DAD collected data from 190 to 400 nm. 

### 2.5. LC-MS Parameters

The tentative identification of the main flavonoids and iridoid glycosides was facilitated by HPLC-DAD-MS. These analyses were performed on an UltiMate 3000 RSLC-series system (Dionex) coupled to an HCT 3D quadrupole ion trap mass spectrometer equipped with an orthogonal ESI source (Bruker Daltonics, Bremen, Germany). HPLC separation was carried out as described above. The eluent flow was split roughly 1 : 8 before the ESI ion source, which was operated as follows: capillary voltage: +3.5/−3.7 kV, nebulizer: 26 psi (N_2_), dry gas flow: 9 L/min (N_2_), and dry temperature: 340°C. The mass spectrometer was operated in an automated data-dependent acquisition (DDA) mode to obtain MS^2^, MS^3^, and MS^4^ spectra (collision gas: He, isolation window: 4 Th, and fragmentation amplitude: 1.0 V).

The identity of the main compounds in the extracts was confirmed either by the comparison of the retention times, UV- and MS^*n*^-spectra, with reference compounds (apigenin, luteolin, chrysoeriol, diosmetin, aucubin, and catalpol), or by isolation and structural characterization by 1D and 2D NMR and MS (melampyroside, mussaenoside, and lunularin). 

### 2.6. NMR Analysis

All 1D (^1^H and ^13^C) and 2D (COSY, NOESY, HMQC, and HMBC) NMR spectra were recorded on a Bruker Avance 500 NMR spectrometer (Bruker BioSpin, Rheinstetten, Germany). The respective compounds were dissolved in methanol-d4 (99.8 atom% D), CD_3_OD [*δ*(^1^H) = 3.31 ppm, and *δ*(^13^C) = 49.00 ppm]. The ^1^H and ^13^C NMR spectra were operated at 500 and 125 MHz, respectively. ^13^C and ^1^H NMR data were consistent with literature values for melampyroside [26, 27] and mussaenoside [27]. Detailed data for melampyroside ([1S-(1*α*,4a*α*,5*α*,7a*α*)]-7-[(benzoyloxy)methyl]-1,4a,5,7a-tetrahydro-5-hydroxycyclopenta[c]pyran-1-yl-*β*-D-glucopyranoside): ^1^H-NMR: (d_4_-methanol, 500 MHz) *δ* 8.07 (2H, Bz H-2, Bz H-6), 7.62 (1H, Bz H-4), 7.49 (2H, Bz H-3, Bz H-5), 6.35 (1H, H-3), 5.86 (1H, H-6), 5.12 (1H, H-4), 5.11, 4.98 (2H, OCH_2_), 5.01 (1H, H-1), 4.70 (1H, H-1′), 4.48 (1H, H-5), 3.84, 3.64 (2H, H-6′), 3.37 (1H, H-3′), 3.28 (1H, H-4′), 3.27 (1H, H-5′), 3.23 (1H, H-2′), 3.00 (1H, H-7a), 2.70 (CH, H-4a). ^13^C-NMR: (d_4_-methanol, 125 MHz) *δ* 167.71 (C, COO), 142.61 (C, C-7), 141.79 (CH, C-3), 134.42 (CH, Bz C-4), 132.78 (CH, C-6), 131.32 (C, Bz C-1), 130.64 (CH, Bz C-2, Bz C-6), 129.68 (CH, Bz C-3, Bz C-5), 105.51 (CH, C-4), 100.17 (CH, C-1′), 97.93 (CH, C-1), 82.88 (CH, C-5), 78.32 (CH, C-5′), 77.97 (CH, C-3′), 74.91 (CH, C-2′), 71.50 (CH, C-4′), 64.07 (CH_2_, OCH_2_), 62.78 (CH_2_, C-6′), 48.56 (CH, C-7a), 46.36 (CH, C-4a). Detailed data for mussaenoside ([1S-(1*α*,4a*α*,7*α*,7a*α*)]-1-(*β*-D-glucopyranosyloxy)-1,4a,5,6,7,7a-hexahydro-7-hydroxy-7-methyl-cyclopenta[c]pyran-4-carboxylic acid methyl ester): ^1^H-NMR: (d_4_-methanol, 500 MHz) *δ* 7.41 (1H, H-3), 5.46 (1H, H-1), 4.67 (1H, H-1′), 3.90, 3.64 (2H, H-6′), 3.71 (3H, OMe), 3.36 (1H, H-3′), 3.32 (1H, H-5′), 3.24 (1H, H-4′), 3.19 (1H, H-2′), 3.17 (1H, H-4a), 2.28, 1.42 (2H, H-5), 2.22 (1H, H-7a), 1,71 (2H, H-6), 1.32 (3H, Me at C-7). ^13^C-NMR: (d_4_-methanol, 125 MHz) *δ* 169.38 (C, COO), 152.04 (C, C-3), 113.38 (C, C-4), 99.81 (CH, C-1′), 95.35 (CH, C-1), 80.51 (C, C-7), 78.40 (CH, C-5′), 78.00 (CH, C-3′), 74.74 (CH, C-2′), 71.71 (CH, C-4′), 62.94 (CH_2_, C-6′), 52.31 (CH, C-7a), 51.64 (CH_3_, OMe), 40.69 (CH_2_, C-6), 32.04 (CH, C-4a), 30.73 (CH_2_, C-6), 24.64 (CH_3_, Me at C-7). The results for lunularin (3′,4-ethylenebisphenol) were as follows: ^1^H-NMR: (d_4_-methanol, 500 MHz) *δ* 7.04 (1H, H-5′), 6.97 (2H, H-2, H-6), 6.67 (2H, H-3, H-5), 6.63 (1H, H-6′), 6.59 (1H, H-2′), 6.58 (1H, H-4′), 2.76 (4H, benzyl CH_2_). ^13^C-NMR: (d_4_-methanol, 125 MHz) *δ* 158.29 (C, C-3′), 156.41 (C, C-4), 144.82 (C, C-1′), 134.02 (C, C-1), 130.37(CH, C-2, C-6), 130.17 (CH, C-5′), 120.85 (C, C-6′), 116.36 (CH, C-2′), 115.99 (CH, C-3, C-5), 113.68 (CH, C-4′), 39.50 (CH_2_, CH_2_ at C-4′), 36.24 (CH_2_, CH_2_ at C-4′).

### 2.7. Interleukin-8 and E-Selectin ELISA

Telomerase reverse transcriptase technology (hTERT) immortalized human umbilical vein endothelial cells (HUVECtert) [28] were cultured in M199 medium supplemented with 20% FCS (both from Sigma-Aldrich, St Louis, USA), cell growth supplement (PromoCell, Germany), and antibiotics. The experiments were performed in sixtuplicate in 96-well plates in M199 medium containing 5% serum. Plant extracts were tested at a concentration of 30 *μ*g/mL, reference pure compounds at 30 *μ*M; BAY 11-7082 was used as positive control (7.5 *μ*M). Subconfluent HUVECtert cells were pretreated for 30 min with the plant material or inhibitor as indicated, followed by stimulation with 10 ng/mL of TNF-*α* (PeproTech, Rocky Hill, USA) or 100 ng/mL of LPS (Sigma-Aldrich, St. Louis, USA) for 6 h. E-selectin and secreted interleukin-8 were determined by whole-cell ELISA or in cell culture supernatants, respectively.

IL-8 ELISA was performed using the Quantikine Human CXCL8/IL-8 Immunoassay Kit (R&D Systems, Minneapolis, USA). Supernatants were transferred into 96-well plates (NALGE-NUNC Int., Rochester, USA) coated with capturing antibody for IL-8 and developed with the respective detection antibody. Peroxidase activity was assessed with TMB 2-Component Microwell Peroxidase Substrate Kit (KPL, Gaithersburg, USA), while the optical density (OD) was measured with a SynergyHT Multi-Detection Microplate Reader (BioTek Instruments, Winooski, USA) at 450 nm using 620 nm wavelength as reference. 

For measuring E-selectin, cells were fixed using 0.1% glutaraldehyde in PBS. E-selectin was detected using 300 ng/mL CD62E mouse antihuman antibody (R&D Systems, Minneapolis, USA) and horseradish peroxidase-linked sheep anti-mouse IgG (GE Healthcare, Waukesha, USA). The same substrate and detection method were used as for IL-8.

### 2.8. NF-*κ*B Transactivation Assay

The transactivation of a NF-*κ*B-driven luciferase reporter gene was quantified in HEK293/NF-*κ*B-luc cells (Panomics, RC0014) as previously described [29]. The cells were maintained at 37°C and 5% CO_2_ cell culture incubators in Dulbecco's modified Eagle's medium (DMEM; Lonza, Basel, Switzerland) supplemented with 2 mM glutamine, 100 *μ*g/mL hygromycin B, 100 U/mL benzylpenicillin, 100 *μ*g/mL streptomycin, and 10% fetal bovine serum (FBS). On the day before the experiment, the cells were stained by incubation for 1 h in serum-free medium supplemented with 2 *μ*M Cell Tracker Green CMFDA (C2925; Invitrogen). The cells were, then, reseeded in 96-well plates at a density of 4 × 10^4^ cells/well in phenol red-free and FBS-free DMEM overnight. Cells were pretreated as indicated for 30 min prior to stimulation with 2 ng/mL TNF-*α* for 4 h. The final concentration of DMSO in the experiments was 0.1% or lower. An equal amount of DMSO was always tested in each experiment to assure that the solvent vehicle does not influence the results. After cell lysis, the luminescence of the firefly luciferase and the fluorescence of the Cell Tracker Green CMFDA were quantified on a Genios Pro plate reader (Tecan, Grödig, Austria). The luciferase-derived signal from the NF-*κ*B reporter was normalized by the Cell Tracker Green CMFDA-derived fluorescence to account for differences in the cell number. The known NF-*κ*B inhibitor parthenolide (Sigma-Aldrich, Vienna, Austria) was used as a positive control.

### 2.9. PPAR Luciferase Reporter Gene Assay

To evaluate PPAR*α* or PPAR*γ* activation, transient transfection of HEK-293 cells with the respective PPAR expression plasmid and a luciferase reporter plasmid containing PPAR response element (PPRE) was used [30]. HEK-293 cells (ATCC, USA) were cultured in DMEM with phenol red, supplemented with 100 U/mL benzylpenicillin, 100 *μ*g/mL streptomycin, 2 mM glutamine, and 10% FBS. The cells were seeded in 10 cm dishes at a density of 6 × 10^6^ cells/dish, incubated for 18 h, and transfected by the calcium phosphate precipitation method with 4 *μ*g of the reporter plasmid (tk-PPREx3-luc), 4 *μ*g PPAR*α* or PPAR*γ* receptor expression plasmid, and 2 *μ*g green fluorescent protein plasmid (pEGFP-N1, Clontech, CA, USA) as internal control. After 6 h, the transfected cells were harvested and reseeded (5 × 10^4^ cells/well) in 96-well plates containing DMEM without phenol red, supplemented with 100 U/mL benzylpenicillin, 100 *μ*g/mL streptomycin, 2 mM glutamine, and 5% charcoal-stripped FBS. The cells were further treated with the indicated extracts or compounds or the solvent vehicle and incubated for 18 h. The medium was, then, discarded, the cells were lysed, and luciferase activity and fluorescence were measured on a Genios Pro plate reader (Tecan, Grödig, Austria). The luminescence signals obtained from the luciferase activity measurements were normalized to the EGFP-derived fluorescence, to account for differences in the transfection efficiency or cell number. The specific PPAR*α* agonist GW7647 (Cayman, Missouri, USA) and the PPAR*γ* agonist pioglitazone (Molekula Ltd., Shaftesbury, UK) were used to verify the subtype specificity of the performed measurements.

### 2.10. Statistical Analysis

Statistical analyses were performed using Prism Software (ver. 4.03; GraphPad Software Inc., San Diego, CA). For NF-*κ*B transactivation and PPAR luciferase reporter gene assay, data were normalized to DMSO treated control the mean value of which was set as 1.0, and for E-selectin and IL-8 expression, the data were analyzed as percent inhibition in comparison to the respective LPS- or TNF-*α*-treatment controls. The experimental data are presented as means ± standard error (SE). Statistical significance was determined by ANOVA using Bonferroni post hoc test. *P* values < 0.05 were considered significant (*P < 0.05, **P < 0.01, ***P < 0.001).

## 3. Results and Discussion

The upregulation of E-selectin and IL-8 production in HUVECtert cells was induced by two diverse stimuli, the proinflammatory cytokine TNF-*α* or the bacterial product LPS. TNF-*α* and LPS activate distinct but partially overlapping signaling pathways implemented in acute and chronic inflammation. They were used as chemically distinct inflammatory agonists interacting with different receptors to enhance the reliance of the screen. Since, next to endothelial cells, the LPS receptor, toll-like receptor 4, and the tumor necrosis factor receptor play a key role in the activation of other cell types, for example, leukocytes, results of the screen are likely to be relevant to other cell types as well. 

The investigations of extracts of *Melampyrum pratense* herb with respect to downregulation of LPS- or TNF-*α*-induced IL-8 and E-selectin expression, PPAR activation, or NF-*κ*B inhibition showed equal or even better results for chlorophyll-depleted and detannified extracts compared to crude DCM and MeOH extracts (Table 1). Further fractionation of these extracts was performed using solid phase extraction. 

The SPE subfractions were further tested for their anti-inflammatory activity *in vitro*. The strongest downregulation of IL-8 and E-selectin induced by LPS or TNF-*α* was found in the 70% SPE fraction of the chlorophyll-free DCM extract as well as in the 30% and 70% SPE subfraction of the detannified MeOH extract (Table 2). Investigations regarding PPAR activation and NF-*κ*B inhibition showed highest activities in the 70% SPE subfractions (Table 2). The high activity of the 100% SPE fractions of the chlorophyll-free DCM extract could be explained by the presence of fatty acids in these fractions that were identified by TLC and GC-MS (data not shown) using reference compounds [31].

**Figure 1:**
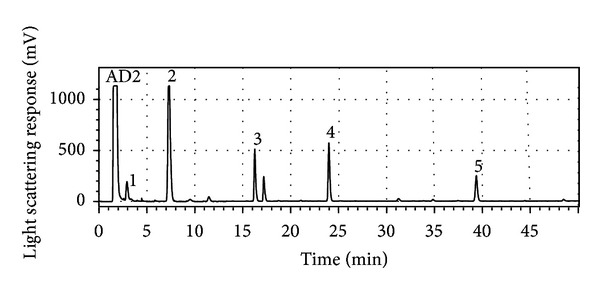
HPLC-ELDS-LT chromatogram of the methanolic extract of *Melampyrum pratense*. Analyses were performed on an Acclaim 120 C_18_ reversed-phase column (150 mm × 2.1 mm i.d., 3 *μ*m) equipped with an Acclaim 120 C_18_ guard column (10 mm × 2.1 mm i.d., 5 *μ*m) from Dionex (Germering, Germany) with the following mobile phase gradient of acetonitrile-water (A: H_2_O + formic acid (pH = 2.5), B: MeCN): 2% B (5 min), 2%–10% of B in 11 min, 10%–20% of B in 24 min, 20%–50% of B in 20 min, and 50%–95% of B in 5 min at a flow rate of 0.4 mL/min and a temperature set to 35°C. The following compounds were identified: (1) catalpol, (2) aucubin, (3) mussaenosidic/loganic acid, (4) mussaenoside, and (5) melampyroside.

HPLC analysis revealed that besides mussaenoside **4**, and melampyroside **5**, the crude methanolic extract contained several other iridoid glycosides, identified as catalpol **1** (shoulder of 2nd peak), aucubin **2**, and mussaenosidic/loganic acid **3** (see Figure 1). The identification of these compounds was done with commercial reference compounds via the comparison of the UV-spectra and retention time as well as by LC-MS analysis.

**Figure 2:**
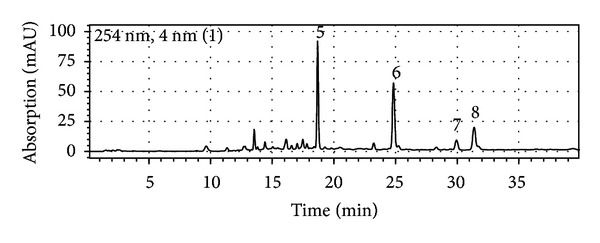
HPLC-DAD (detection wavelength 254 nm) chromatogram of the detannified MeOH extract of *Melampyrum pratense*. The chromatographic separation was assessed on an Acclaim 120 C_18_ reversed-phase column (150 mm × 2.1 mm i.d., 3 *μ*m) equipped with an Acclaim 120 C_18_ guard column (10 mm × 2.1 mm i.d., 5 *μ*m) from Dionex (Germering, Germany) using the following mobile phase gradient of acetonitrile-water (A: H_2_O + formic acid (pH = 2.5), B: MeCN): 2% B (5 min), in 5 min to 15% B, in 3 min to 21% B, in 27 min to 35% B, and in 10 min to 95% B at a flow rate of 0.4 mL/min and a temperature set to 35°C. The following compounds could be identified: (5) melampyroside, (6) luteolin, (7) apigenin, and (8) chrysoeriol.

On the other hand, the detannified MeOH extract mainly contained flavonoids of which luteolin **6**, apigenin **7**, chrysoeriol **8**, and traces of diosmetin could be identified (Figure 2) by comparison of the UV-spectra and retention time with commercial reference standards as well as a comparison with an MS flavonoid database [32].

**Figure 3:**
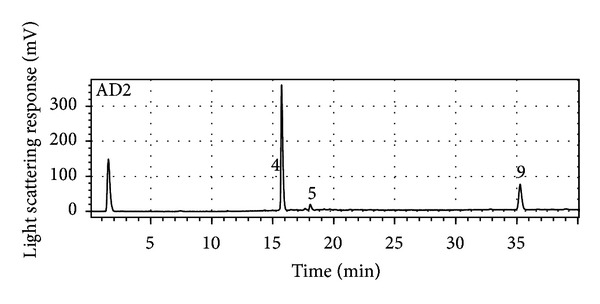
HPLC-ELDS-LT chromatogram of the chlorophyll-depleted dichloromethane extract of *Melampyrum pratense*. Analyses were performed on an Acclaim 120 C_18_ reversed-phase column (150 mm × 2.1 mm i.d., 3 *μ*m) equipped with an Acclaim 120 C_18_ guard column (10 mm × 2.1 mm i.d., 5 *μ*m) from Dionex (Germering, Germany) with the following mobile phase gradient of acetonitrile-water (A: H_2_O + formic acid (pH = 2.5), B: MeCN): 2% B (5 min), in 5 min to 15% B, in 3 min to 21% B, in 27 min to 35% B, and in 10 min to 95% B at a flow rate of 0.4 mL/min and a temperature set to 35°C. The following compounds were identified: (4) mussaenoside, (5) melampyroside, and (9) lunularin.

The chlorophyll-free DCM extract mainly contained mussaenoside, next to lunularin **9** and melampyroside (Figure 3). Mussaenoside and melampyroside were enriched in the 30% SPE subfraction. The main component of the 70% SPE subfraction was lunularin. The identification was validated via the comparison of retention times as well as UV-spectra with those of the isolated purified reference compounds (structure confirmed by NMR and LC-MS).

**Figure 4:**
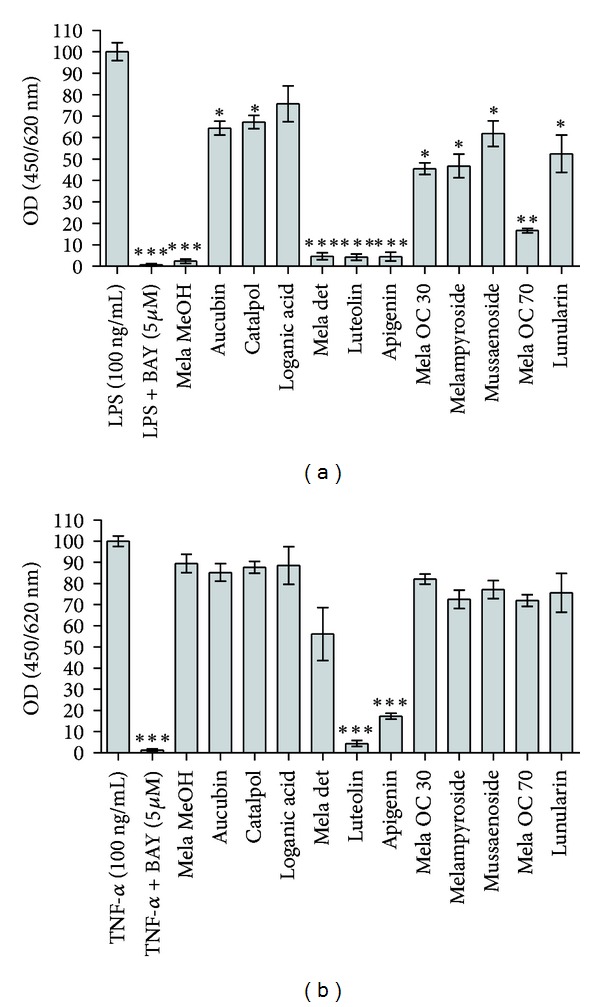
Effect of *Melampyrum pratense *extracts and their individual components on the induction of E-selectin protein after stimulation with LPS (a) or TNF-*α* (b). Mela MeOH: methanolic extract, Mela det: detannified methanol extract, and Mela OC 30/OC 70: 30%/70% methanolic SPE subfraction of the chlorophyll-depleted dichloromethane extract. Extracts and fractions were tested at 30 *μ*g/mL, compounds at 30 *μ*M. BAY (5 *μ*M) was used as a positive control. The data are presented as mean values ± standard errors of sixtuplicate measurements. Very similar results were obtained in an independent experiment. Stars indicate statistical significance compared to LPS or TNF-*α* activation (ANOVA): *P < 0.05, **P < 0.01, and ***P < 0.001.

**Figure 5:**
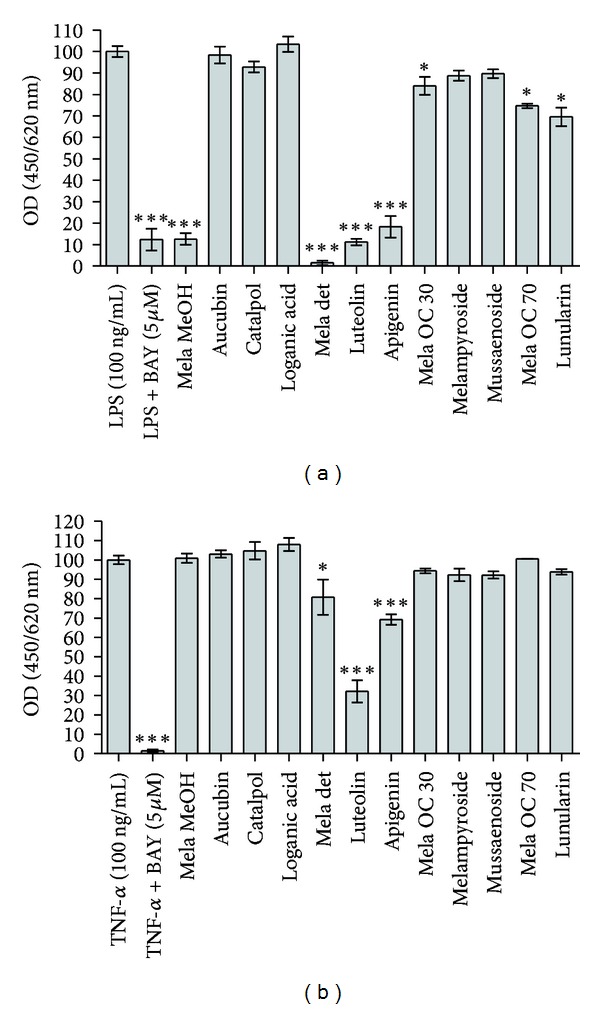
Effect of *Melampyrum pratense *extracts and their individual components on the induction of IL-8 protein after stimulation with LPS (a) or TNF-*α* (b). Mela MeOH: methanolic extract, Mela det: detannified methanol extract, and Mela OC 30/OC 70: 30%/70% methanolic SPE subfraction of the chlorophyll-depleted dichloromethane extract. Extracts and fractions were tested at 30 *μ*g/mL, compounds at 30 *μ*M. BAY (5 *μ*M) was used as a positive control. The data are presented as mean values ± standard errors of sixtuplicate measurements. Very similar results were obtained in an independent experiment. Stars indicate statistical significance compared to LPS or TNF-*α* activation (ANOVA): *P < 0.05, **P < 0.01, and ***P < 0.001.

**Figure 6:**
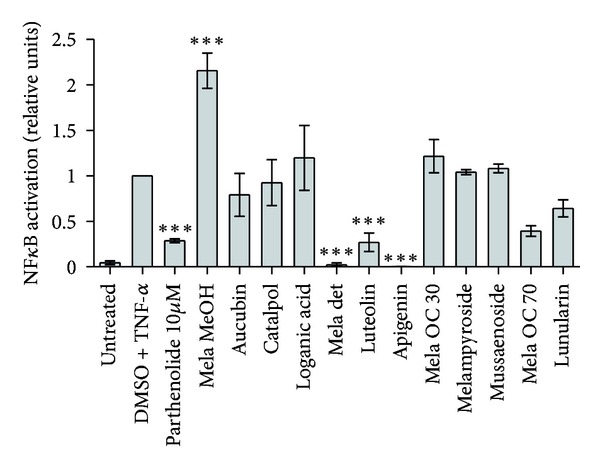
Influence of *Melampyrum pratense* extracts (Mela MeOH: methanolic extract; Mela det: detannified methanol extract), subfractions (Mela OC 30/OC 70: 30%/70% methanolic SPE subfraction of the chlorophyll-depleted dichloromethane extract) and the respective compounds thereof on NF-*κ*B activation. Extracts and fractions were tested at a concentration of 30 *µ*g/mL, compounds at 30 *μ*M with TNF-*α* cotreatment, in quadruplicate in three independent experiments. Parthenolide (10 *μ*M) was used as positive control. Bars represent mean values, error bars consider standard error, and stars indicate significance compared to TNF-*α* induction (ANOVA/Bonferroni): ***P < 0.001.

The main compounds from *M. pratense* were tested together with their initial extract for PPAR activation, NF-*κ*B inhibition, and the downregulation of LPS- or TNF-*α*-induced production of IL-8 and E-selectin. The iridoid glycosides aucubin, catalpol, melampyroside, and mussaenoside reduced E-selectin expression in response to LPS stimulation (Figure 4). The effect of iridoidglycosides was specific for E-selectin as they did not downregulate IL-8 (Figure 5) nor activate PPARs (data not shown) or inhibit NF-*κ*B (Figure 6). The iridoids catalpol and aucubin can be found in many plant families and have already been described for *M. pratense* and other *Melampyrum* species [24, 33]. Jeong et al. (2002) showed that aucubin inhibited the expression of TNF-*α* and IL-6 in Ag-stimulated rat basophilic leukemia- (RBL-) 2H3 mast cells in a dose-dependent manner with an IC_50_ of 0.10 and 0.19 *μ*g/mL, respectively. In that study, aucubin was also determined to inhibit antigen-induced nuclear translocation of the p65 subunit of NF-*κ*B. Since activator protein-1 binding activity was not affected, its results suggested that aucubin is a specific inhibitor of NF-*κ*B activation in mast cells, which might explain its beneficial effect in the treatment of chronic inflammatory diseases [34]. However, in our experimental setup, aucubin did not inhibit NF-*κ*B at 30 *μ*M.

The flavonoids apigenin and luteolin, on the other hand, showed strong inhibition of NF-*κ*B (Figure 6) as well as a strong downregulation of both IL-8 and E-selectin proteins (Figures 4 and 5). Since luteolin and apigenin displayed the highest activity on all anti-inflammatory targets, dose-response studies were performed. Luteolin downregulated E-selectin with an IC_50_ of 5.67 *μ*M in TNF-*α*-, and an IC_50_ of 5.10 *μ*M in LPS-stimulated endothelial cells. IL-8, on the other hand, was downregulated with an IC_50_ of 32.1 *μ*M and 27.1 *μ*M, respectively. Apigenin showed significant inhibition of both E-selectin and IL-8. An IC_50_ of 31.7 *μ*M in TNF-*α*- and 18.9 *μ*M in LPS-stimulated cells was observed concerning IL-8 expression. E-selectin expression was inhibited with an IC_50_ of 17.7 *μ*M after stimulation with TNF-*α* and 13.6 *μ*M after stimulation with LPS. For flavonoids in general, inhibitory effects on numerous inflammatory targets or signaling pathways in the micromolar concentration range have been reported. The NF-*κ*B inhibitory effect of apigenin and luteolin was detected in our study by an NF-*κ*B-responsive luciferase reporter assay (Figure 6) and by ELISA monitoring the expression of the NF-*κ*B target genes E-selectin and IL-8 (Figures 4 and 5). A number of previous studies have also demonstrated the NF-*κ*B inhibitory action of these two flavonoids utilizing a number of different methods and cell models [35–41]. Depending on little changes in the flavone backbone, flavonoids can play a modulating, biphasic, and regulatory action on immunity and inflammation [42]. Studies regarding the effect of flavonols (kaempferol, quercetin, and myricetin) and flavones (flavone, chrysin, apigenin, luteolin, baicalein, and baicalin) on TNF-*α*-stimulated ICAM-1 expression, which is like E-selectin a proinflammatory adhesion molecule, revealed kaempferol, chrysin, apigenin, and luteolin as active inhibitors of ICAM-1 expression. Additionally, apigenin and luteolin were shown to inhibit the NF-*κ*B signaling at the level of I*κ*B kinase (IKK) activity, with consequent effect on I*κ*B*α* degradation, NF-*κ*B binding to the DNA, and NF-*κ*B transactivation activity [35]. Several flavonoids were investigated for the inhibitory effect on LPS-induced TNF-*α* production from macrophages. Positive results were provided for the flavones luteolin, apigenin, and chrysin, the flavonols quercetin and myricetin, the flavanonol taxifolin, and the anthocyanidin cyanidin chloride *in vitro*. Nevertheless, serum TNF-*α* production *in vivo* was inhibited only by luteolin or apigenin, and only luteolin or quercetin inhibited 12-O-tetradecanoylphorbol-13-acetate (TPA) induced ear edema. These results suggest that the structure of luteolin is the most suitable for the oral anti-inflammatory activity and that the presence of absence of a hydroxy group may cause a loss of efficiency [43]. Moreover, luteolin was shown to display excellent radical scavenging and cytoprotective properties, especially when tested in complex biological systems where it can interact with other antioxidants like vitamins. Nevertheless, its anti-inflammatory effects at micromolar concentrations can only be partly explained by its antioxidant capacities. They include the activation of antioxidative enzymes, suppression of the NF-*κ*B pathway, and inhibition of proinflammatory substances. The glycosidic form of luteolin often present in plants is cleaved *in vivo*, and the aglycones are conjugated and metabolized after nutritional uptake which has to be considered when evaluating *in vitro *studies [44]. Besides the inhibition of the induction of inflammatory cytokines such as TNF-*α*, IL-8, IL-6 and granulocyte-macrophage colony-stimulating factor (GM-CSF), luteolin was found to attenuate cyclooxygenase (COX)-2 expression and rise in intracellular Ca^2+^ levels [45]. In our study, the stilbene-like compound lunularin only had an inhibitory effect on IL-8 and E-selectin after stimulation with LPS but not with TNF-*α*. This compound is mainly known from liverworts [46, 47]. Besides, in higher plants, it was found in celery, *Apium graveolens* [48], and *Morus* species [49]. Activity studies have only been conducted concerning antibacterial, antifungal, and antioxidant characteristics [50], but the anti-inflammatory effects of lunularin have not yet been reported. Thus, by showing its ability to downregulate E-selectin and IL-8 in endothelial cells stimulated with LPS, the present study gives first insight into a possible anti-inflammatory action of this compound.

## 4. Conclusion

The extracts of *Melampyrum pratense* were found to decrease the production of inflammatory proteins E-selectin and IL-8 in HUVECtert cells *in vitro*. Using bioactivity-guided fractionation and HPLC-ELSD/-DAD/-MS structure elucidations several iridoids and flavonoids were isolated and identified as potential active principles. Melampyroside, mussaenoside, and lunularin were identified in *M. pratense* for the first time. The isolated iridoid glycosides mussaenoside and melampyroside were able to downregulate E-selectin, especially after stimulation with LPS, suggesting them to act on this specific pathway. The flavonoids apigenin and luteolin, on the other hand, showed strong inhibition of NF-*κ*B as well as strong downregulation of both IL-8 and E-selectin and, thus, were considered the main constituents responsible for the anti-inflammatory activity of the plant. The stilbene-like compound lunularin only inhibited E-selectin and IL-8 expression after stimulation with LPS not with TNF-*α*. Additionally, this compound did slightly activate PPAR*γ* (data not shown). Aside from lunularin, the only further PPAR*α* and PPAR*γ* ligands that we identified in the plant extracts were different fatty acids, which represent well-known PPAR activators [31] and, thus, were not further investigated.

Finally, the present investigations contribute to the understanding of the chemical composition in relation to the anti-inflammatory effects elicited by *Melampyrum pratense* extracts.

## Figures and Tables

**Table 1:** Investigation of *Melampyrum pratense* extracts for agonistic activity towards PPAR*α* and PPAR*γ*, inhibition of TNF-*α* induced NF-*κ*B activity, and reduction of TNF-*α* or LPS induced expression of IL-8, and E-selectin *in vitro*.

Plant material	Preparation	PPAR*α* activation	PPAR*γ* activation	NF-*κ*B inhibition	E-selectin +TNF	IL-8 +TNF	E-selectin +LPS	IL-8 +LPS
*Melampyrum pratense *herb	DCM extract	−	−	−	+++	+	++	+++
	DCM extract chlorophyll-depleted	−	−	−	++	+++	++	+++
	MeOH extract	−	−	−	+++	+++	+++	+++
	MeOH extract detannified	−	−	+++	+++	+++	++	+++

DCM: dichloromethane; MeOH: methanol. Dry material from all test samples was dissolved in DMSO at a concentration of 10 mg/mL and tested at a final concentration of 10 *μ*g/mL. Regarding PPARs, no activation compared to control (0.1% DMSO): −; Regarding NF-*κ*B, IL-8, and E-selectin: inhibition in the range of 0%–25% compared to the vehicle treated control (0.1% DMSO): −; 25%–50% inhibition (*P* < 0.05): +; 50%–80% inhibition (*P* < 0.05): ++; over 80% inhibition (*P* < 0.05): +++. At least three independent experiments were performed with every sample, and statistical analysis was performed by ANOVA.

**Table 2:** Results of the SPE subfractions of *Melampyrum pratense* chlorophyll-depleted DCM extract and detannified MeOH extract tested on inflammatory targets *in vitro*.

Initial extract	Preparation	PPAR*α* activation	PPAR*γ* activation	NF-*κ*B inhibition	E-selectin +TNF	IL-8 +TNF	E-selectin +LPS	IL-8 +LPS
DCM extract chlorophyll-depleted	SPE 30% MeOH	−	+	+	++	−	++	−
	SPE 70% MeOH	+++	+++	+++	+++	−	+++	+++
	SPE 100% MeOH	+++	+++	+++	−	−	++	++
MeOH extract detannified	SPE 30% MeOH	−	+	+++	++	−	++	+++
	SPE 70% MeOH	+++	+++	+++	+++	−	++	++
	SPE 100% MeOH	−	+	+	−	−	+++	+++

Fractions were tested in triplicates at a concentration of 10 *µ*g/mL for PPARs activation, TNF-*α* induced NF-*κ*B inhibition, and reduction of LPS or TNF-*α* induced expression of IL-8 and E-selectin. Regarding PPARs, no activation compared to control (0.1% DMSO): −; 1.25 to 1.5-fold activity compared to control (*P* < 0.05): +; 1.5 to 2-fold activity compared to control (*P* < 0.05): ++; more than 2-fold of the control (*P* < 0.05): +++. Regarding NF-*κ*B, IL-8, and E-selectin, inhibition in the range of 0%–25% compared to the vehicle treated control (0.1% DMSO): −; 25%–50% inhibition (*P* < 0.05): +; 50%–80% inhibition (*P* < 0.05): ++; over 80% inhibition (*P* < 0.05): +++. At least three independent experiments were performed with every sample, and statistical analysis was performed by ANOVA.

## Abbreviations

ASE: Accelerated solvent extractor
CC: Column chromatography
DCM: Dichloromethane
Det: Detannified methanol extract
DMEM: Dulbecco's modified Eagle's medium
DMSO: Dimethylsulfoxide
EGFP: Enhanced green fluorescent protein
ESIMS: Electrospray ionization mass spectrometry
EtOAc: Ethyl acetate
FBS: Fetal bovine serum
GC-MS: Gas chromatography mass spectrometry
IKK: I*κ*B kinase
IL: Interleukin
LPS: Lipopolysaccharide
MeCN: Acetonitrile
MeOH: Methanol
NF-*κ*B: Nuclear factor *κ*B
NMR: Nuclear magnetic resonance
OC: Chlorophyll-depleted dichloromethane extract
PPAR: Peroxisome proliferator-activated receptor
PPREs: PPAR response elements
SPE: Solid phase extraction
TLC: Thin layer chromatography
TNF: Tumor necrosis factor.
